# Genomic Characterisation of Small Cell Lung Cancer Patient-Derived Xenografts Generated from Endobronchial Ultrasound-Guided Transbronchial Needle Aspiration Specimens

**DOI:** 10.1371/journal.pone.0106862

**Published:** 2014-09-05

**Authors:** Tracy L. Leong, Kieren D. Marini, Fernando J. Rossello, Samantha N. Jayasekara, Prudence A. Russell, Zdenka Prodanovic, Beena Kumar, Vinod Ganju, Muhammad Alamgeer, Louis B. Irving, Daniel P. Steinfort, Craig D. Peacock, Jason E. Cain, Anette Szczepny, D. Neil Watkins

**Affiliations:** 1 MIMR-PHI Institute, Clayton, Victoria, Australia; 2 Monash University, Clayton, Victoria, Australia; 3 Life Sciences Computation Centre, Victorian Life Sciences Computation Initiative, Carlton, Victoria, Australia; 4 Department of Anatomical Pathology, St Vincent's Hospital, Fitzroy, Melbourne, Victoria, Australia; 5 Department of Pathology, Monash Health, Clayton, Victoria, Australia; 6 Department of Medical Oncology, Monash Health, East Bentleigh, Victoria, Australia; 7 Department of Respiratory Medicine, Royal Melbourne Hospital, Parkville, Victoria, Australia; 8 Translational Hematology and Oncology Research, Cleveland Clinic, Cleveland, Ohio, United States of America; 9 The Kinghorn Cancer Centre, Garvan Institute of Medical Research, Darlinghurst, New South Wales, Australia; Memorial Sloan-Kettering Cancer Center, United States of America

## Abstract

Patient-derived xenograft (PDX) models generated from surgical specimens are gaining popularity as preclinical models of cancer. However, establishment of PDX lines from small cell lung cancer (SCLC) patients is difficult due to very limited amount of available biopsy material. We asked whether SCLC cells obtained from endobronchial ultrasound-guided transbronchial needle aspiration (EBUS-TBNA) could generate PDX lines that maintained the phenotypic and genetic characteristics of the primary tumor. Following successful EBUS-TBNA sampling for diagnostic purposes, we obtained an extra sample for cytologic analysis and implantation into the flanks of immunodeficient mice. Animals were monitored for engraftment for up to 6 months. Histopathologic and immunohistochemical analysis, and targeted next-generation re-sequencing, were then performed in both the primary sample and the derivative PDX line. A total of 12 patients were enrolled in the study. EBUS-TBNA aspirates yielded large numbers of viable tumor cells sufficient to inject between 18,750 and 1,487,000 cells per flank, and to yield microgram quantities of high-quality DNA. Of these, samples from 10 patients generated xenografts (engraftment rate 83%) with a mean latency of 104 days (range 63–188). All but one maintained a typical SCLC phenotype that closely matched the original sample. Identical mutations that are characteristic of SCLC were identified in both the primary sample and xenograft line. EBUS-TBNA has the potential to be a powerful tool in the development of new targeting strategies for SCLC patients by providing large numbers of viable tumor cells suitable for both xenografting and complex genomic analysis.

## Introduction

Small cell lung cancer (SCLC) accounts for approximately 15% of all thoracic malignancies [1]. Patients with disease confined to the chest are treated with chemo-radiotherapy, whereas patients with advanced disease are treated with chemotherapy alone [2]. In advanced disease, platinum-based doublet chemotherapy induces complete responses in up to 20%, whereas combined chemo-radiotherapy in disease limited to the chest produces complete responses in up to 50% of patients [3]. However, lethal recurrences within 12 months occur in almost all cases. In addition, trials of multiple cytotoxic agents, dose intensification, or novel targeted therapies have failed to improve outcome over the last three decades [3].

Accurate preclinical models and high quality tissue samples are essential for the development of new cancer therapies. Since surgical resection of SCLC is uncommon, diagnosis and biomarker studies rely heavily on samples obtained by percutaneous fine needle aspiration or bronchoscopic forceps biopsy [1]. Both techniques provide precious little material for researchers, leading to a heavy reliance on conventional cell lines that may not accurately reflect the complex biological and genomic heterogeneity of the human disease [4]. More recently, PDX models have gained popularity amongst cancer researchers. Here, tissue obtained from fresh surgical specimens can be implanted into immunodeficient mice and maintained as an unlimited source of tumor material that closely resembles the primary tumor [4]–[7]. In addition, “co-clinical” trials are now possible, where patient and mouse receive the same therapy [8]. However, the limited availability of high-quality SCLC tissue makes such an approach extremely challenging [4].

EBUS-TBNA is a new development in diagnostic bronchoscopy that permits highly accurate aspiration sampling of tumors and lymph nodes adjacent to the airway that are not visible by conventional bronchoscopy [9]. Since SCLC is commonly associated with mediastinal lymphadenopathy [1], EBUS-TBNA is an ideal way of obtaining material for diagnosis and staging. Therefore, we tested the feasibility of using live cells obtained from EBUS-TBNA sampling to generate PDX lines from SCLC patients.

## Materials and Methods

### Ethics

Human experimentation was undertaken with informed, written consent in accordance with the policies of the National Health and Medical Research Council of Australia, Monash Health, Melbourne Health and the Declaration of Helsinki. The clinical and research protocols were approved by an Australian Multisite Human Research Ethics Committee (Protocol #HREC/12/SHA/8). Animal experimentation was approved by the Monash University Animal Ethics Committee (Protocol #09072A), and was performed in accordance with the National Health and Medical Research Council of Australia and the National Research Council. Humane killing of mice was performed using inhaled carbon dioxide anaesthesia in accordance with institutional and national guidelines.

### Patients and study design

Patients with suspected lung cancer undergoing EBUS-TBNA as part of routine clinical care gave informed consent for one extra biopsy to be taken for research purposes. Those that were found to have SCLC were then included for the analysis presented below.

### EBUS-TBNA

All procedures were carried out as outpatient procedures as previously described [10], in accordance with British Thoracic Society guidelines [11], under conscious sedation together with topical airway anaesthesia using lignocaine 2%. All procedures were carried out using a dedicated linear array bronchoscope (BF-UC180F-OL8, Olympus, Tokyo, Japan). Ultrasound images were processed by a dedicated Doppler-mode ultrasound scanner (EU-ME1, Olympus, Tokyo, Japan).

The first specimen was obtained according to standard clinical protocols. The initial 3 drops of the aspirate was recovered onto a positively charged slide, air dried, and then simultaneously fixed and stained using the Diff-Quik protocol and assessed on-site by a cytopathologist. The remainder of the specimen was then placed in a sterile solution and transported to the diagnostic pathology laboratory where it is centrifuged, fixed in formalin, embedded in paraffin and sectioned for routine histopathology and immunohistochemical analysis. Once the on-site diagnosis was confirmed, a second specimen was then taken for research purposes.

### Processing of research EBUS-TBNA specimen

The entire research specimen was recovered in a sterile 1.5 ml Eppendorf tube, placed on wet ice, and then transported immediately to the laboratory. Ice-cold, sterile phosphate buffered saline (PBS) was added to the specimen in order to make a total volume of 500 µl, and then centrifuged at 1000 g for 5 seconds. The specimen was then gently mixed with a 1 ml pipette and placed on ice. The specimen is then divided as follows: (i) 200 µl was transferred to a Nunc Cryotube and stored at -80°C for subsequent DNA purification; (ii) 50 µl was smeared on a positively charged slide, air dried and then stained using the Diff Quik protocol to determine tumour cell purity; (iii) 50 µl was transferred to a fresh Eppendorf tube, and vigorously triturated with a 200 µl pipette to mechanically disaggregate the cells followed by counting using a hemocytometer to determine total cell number; (iv) the remaining 200 µl was used to generate the PDX. The total number of tumor cells injected was estimated by determining the number tumor cells seen in the Diff-Quick stained slides as percentage of all nucleated cells by averaging the counts of 10 random fields at 40X.

### Generation of PDX lines

The EBUS-TBNA sample was mixed with an equal volume of ice cold Matrigel (BD Biosciences), placed in a sterile 1 ml syringe capped with a 26 G needle and transported immediately to the animal facility. Under aseptic conditions, the cell suspension was injected subcutaneously into the right flank of a NSG mouse (NOD.Cg-Prkdcscid Il2rgtm1Wjl/SzJ). These profoundly immunodeficient animals are derived from the NOD/SCID strain with the addition of a homozygous Interleukin-2 receptor gamma chain knockout, and are maximally efficient at establishing xenograft tumors from a small number of donor cells [12]. Once primary engraftment was achieved, PDX lines were then passaged in nude mice as previously described [4].

### Immunohistochemistry

Staining was performed on 5 µm formalin fixed, paraffin embedded sections as described [13], using the Vectastain Elite ABC Kit (Vector Laboratories; PK-6101) and the Mouse on Mouse Basic Kit (Vector Laboratories; BMK-2202). Antibodies and dilutions were as follows: rabbit polyclonal anti-NCAM (CD56) a neural and neuroendocrine marker,(H-300) (Santa Cruz Biotechnology; sc-10735), 1∶200; rabbit monoclonal anti-TTF1 (EP1584Y,Novus Biologicals; NB100-8006), 1∶400; mouse monoclonal anti-Synaptophysin (Ventana Clone SP11, pre-diluted).

### Targeted re-sequencing

Snap frozen EBUS and PDX samples were used to generate purified DNA using the Qiagen DNeasy kit (#69504) with the Qiagen RNAse A treatment option (#19101) according to the manufacturer's instructions. DNA was assayed and quality controlled using the Qubit 2.0 Fluorimeter (Invitrogen #Q32866) according to the manufacturer's instructions.

Targeted re-sequencing was performed using the Ion AmpliSeq comprehensive cancer panel v2 (Life Technologies #4477685), which targets exons of 409 tumor suppressor genes and oncogenes. Library construction was performed using the Ion AmpliSeq Library Kit 2.0 (Life Technologies, #4478379) and library templates were prepared and barcoded for sequencing using the Ion OneTouch System as per manufacturer's instructions. Four barcoded samples were multiplexed per Ion PI Chip (Life Technologies) and sequenced on the Ion Proton Sequencer System (Life Technologies). Sequencing reads were processed using Ion Torrent Suite software v 4.0.2 (Life Technologies). De-multiplexed samples were assessed for sequencing quality and high quality sequencing reads were mapped to the complete hg19 human genome (UCSC version, February 2009). Variant discovery was performed using Torrent Variant Caller v 4.0 (Life Technologies), a software plug in for the Ion Torrent Suite software. Sample identified variants were pooled and compared using VcfTools [14] and SnpSift [15]. Variant functional annotation was performed using SnpEff [15], and SnpSift with dbNSFP [16], a database for functional annotation of non-synonymous variants. All data and metadata are available at the NIH Short Reads Archive, (www.ncbi.nlm.nih.gov/sra), accession number SRP044662.

## Results

### Patient and sample characteristics

A total of 12 patients with a confirmed diagnosis of SCLC were entered into the study, and are summarised in Table 1. EBUS samples were taken from defined nodal stations, or from large masses in the paratracheal, hilar or mediastinal regions. All samples were assessed using routine cytopathology criteria as being consistent with a diagnosis of SCLC. Where available, cell blocks were sectioned and H&E stained sections confirmed the diagnosis. Immunohistochemical staining of cell block sections was performed for Synaptophysin, CD56 and Thyroid Transcription Factor 1 (TTF1) in 8, 2 and 1 cases respectively.

**Table 1:** Patient and sample characteristics.

Patient Information						Clinical Sample
*ID*	*Age*	*Sex*	*Smoker*	*Stage*	*EBUS Sample*	*Cytology*	*H&E*	*Synapto*	*CD56*	*TTF1*
LX101	75	M	Former	IV	4R	SCLC	SCLC	+	+	N/A
LX102	72	M	Former	IIIA	R Paratracheal	SCLC	SCLC	+	N/A	N/A
LX103	65	F	Former	IIA	12R	SCLC	SCLC	+	N/A	N/A
LX104	65	M	Current	IIIB	4R	SCLC	SCLC	N/A	+	N/A
LX105	56	M	Former	IV	L Paratracheal	SCLC	N/A	N/A	N/A	N/A
LX106	55	M	Current	IV	4L	SCLC	SCLC	+	N/A	N/A
LX107	66	F	Current	IV	4R	SCLC	SCLC	+	N/A	N/A
LX108	59	F	Former	IV	7	SCLC	SCLC	+	N/A	N/A
LX109	72	F	Current	IV	4R	SCLC	SCLC	+	N/A	N/A
LX110	69	M	Current	IIIA	10R	SCLC	SCLC	+	N/A	+
LX111	60	F	Former	IV	7	SCLC	N/A	N/A	N/A	N/A
LX112	59	F	Former	IV	7	SCLC	N/A	N/A	N/A	N/A

SCLC  =  small cell lung cancer; N/A  =  not available; H&E  =  section stained with haematoxylin and eosin; Results of cell block sections stained with antibodies against Synaptophysin (Synapto), CD56 and Thyroid Transcription Factor 1 (TTF1) are also shown. Lymph node stations are indicated according to the classification of Mountain & Dressler [36].

### Generation of PDX lines

Tumor cells were recovered in the research specimen in 12 consecutive cases, and were analysed and implanted into the flanks of NSG mice as described above. The number of tumor cells injected ranged from between 18,750 and 1,487,000 cells per flank. DNA extraction was then retrospectively performed on the 10 samples that successfully generated xenografts using frozen aliquots of the same number of cells as were used to establish the PDX. This yielded between 0.31µg and 11.12 µg high quality genomic DNA suitable for next-generation sequencing analysis.

First passage PDX tumors appeared between 63 and 188 days following implantation (mean 104 days). Following engraftment and growth to a size of 800 mm^3^, the first passage recipient mouse was sacrificed, the tumor dissected from the associated skin and muscle, and a formal mouse necropsy performed. No metastatic lesions were identified in any of the animals. Fresh tumor samples (4–5 mm^3^) were taken and snap frozen for subsequent DNA extraction, and a further sample was fixed in formalin and sectioned for histopathologic and immunohistochemical analysis. The remaining tumor was then mechanically disaggregated, and 1×10^6^ cells re-implanted in the flanks of 5 new recipient nude athymic mice. Once these tumors reached a size of 800 mm^3^, the tumor was sampled in an identical fashion, and 1×10^6^ aliquots were then cryopreserved.

**Figure 1:**
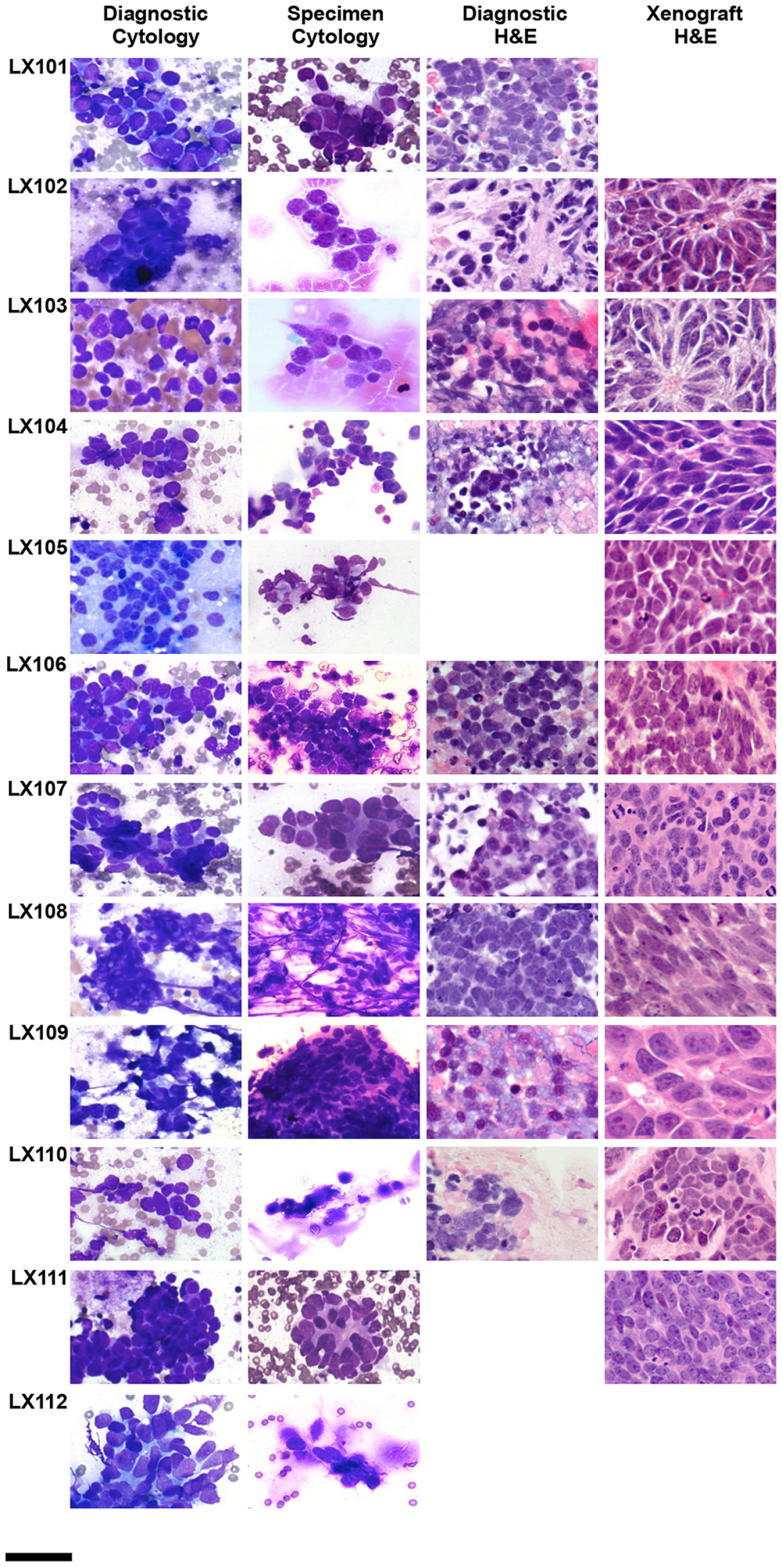
Cytopathology and histopathology analysis of EBUS-TBNA diagnostic and research specimens and derivative xenograft. Scale bar = 30 µm. H&E  =  haemotoxylin and eosin stain. Blank squares indicate that the sample was not available.

**Figure 2:**
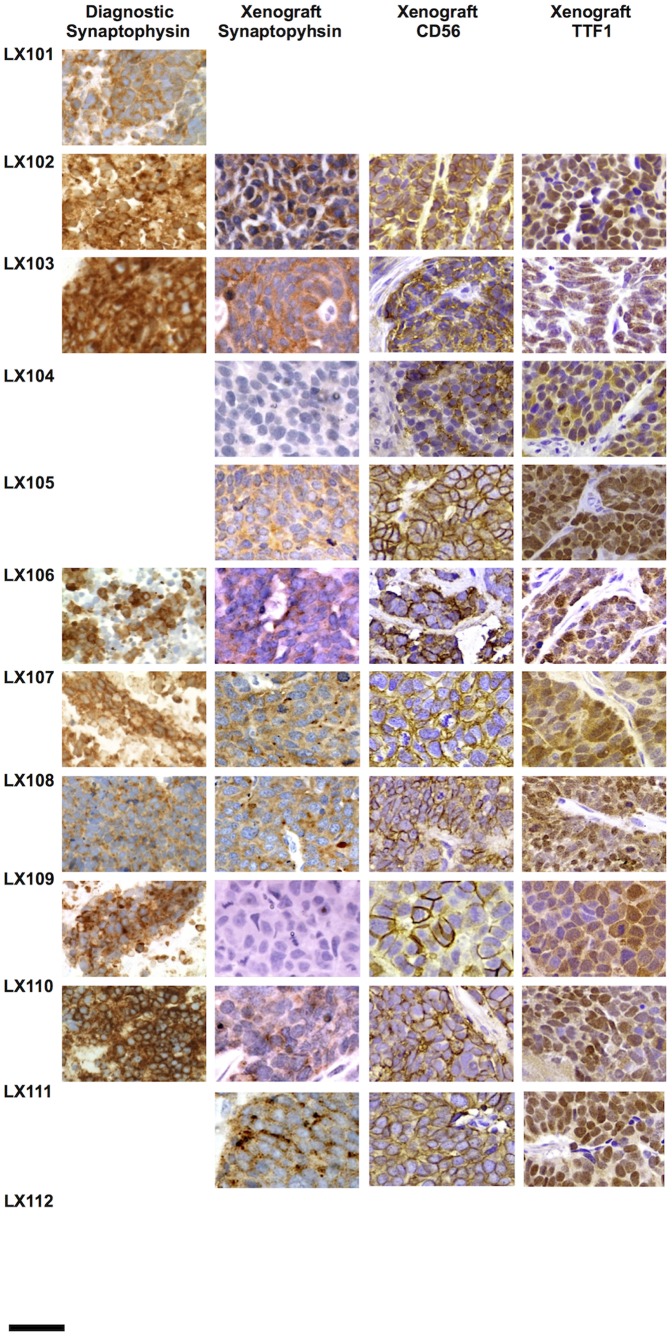
Immunohistochemical analysis of EBUS-TBNA diagnostic and research specimens and derivative xenografts. Scale bar = 30 µm. Blank squares indicate that the sample was not available.

**Figure 3:**
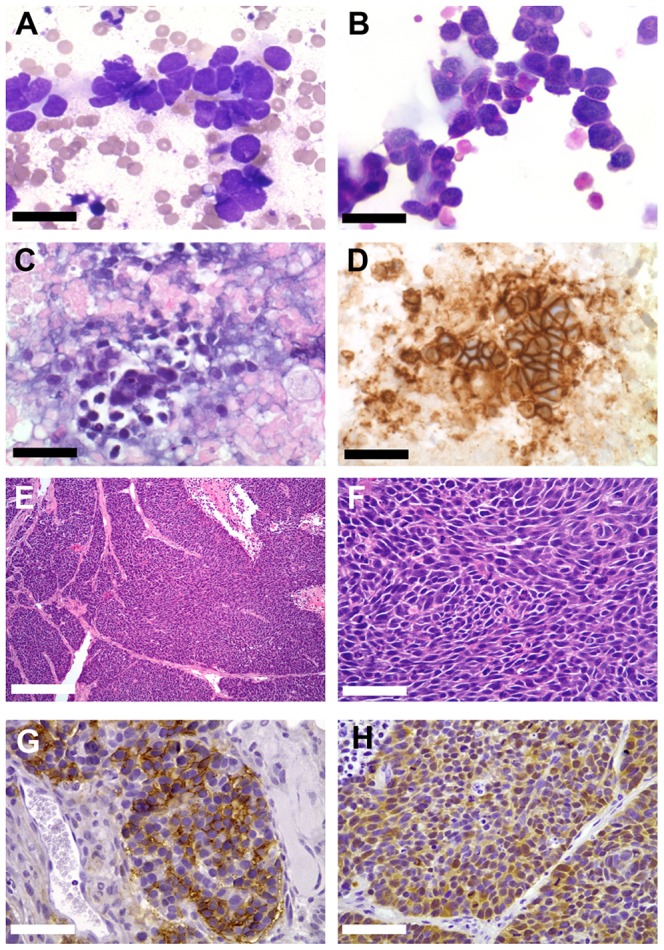
Features of specimen LX104 and its derivative xenograft. **A.** Diff-Quick stained cytology smear of diagnostic EBUS-TBNA sample. Scale bar = 15 µm. **B.** Diff-Quick stained cytology smear of experimental EBUS-TBNA sample. Scale bar = 15 µm. **C.** Haematoxylin and eosin stained section of the diagnostic cell block. Scale bar = 30 µm. **D.** Diagnostic cell block stained for CD56. Scale bar = 30 µm. **E.** Haematoxylin and eosin stained section of the derivative xenograft. Scale bar = 300 µm. **F.** Haematoxylin and eosin stained section of the derivative xenograft. Scale bar = 30 µm. **G.** Section of the derivative xenograft stained for CD56. Scale bar = 30 µm. **H.** Section of the derivative xenograft stained for TTF1. Scale bar = 30 µm.

Of the 10 successful grafts, 9 retained a typical SCLC phenotype, as well as expressing typical immunohistochemical markers of a malignant neuroendocrine tumor at passage 1 and 2. Representative images from the entire sample set are shown in Figures 1 and 2, and a detailed example is shown in Figure 3. One PDX line, LX109, exhibited features consistent with a large cell neuroendocrine tumor, including larger nuclei with distinct nucleoli, prominent eosinophilic cytoplasm, and patchy staining for CD56 (Figure S1). With limited diagnostic material available to review, we are unable to determine if this represent outgrowth of a subpopulation of large cell tumor cells from the experimental specimen. A summary of these data is shown in Table 2.

**Table 2:** Research sample and PDX characteristics.

Research Sample						PDX			
*ID*	*Purity*	*Tumor Cells Injected*	*Xenograft*	*Latency (Days)*	*DNA µg)*	*H&E*	*Synapto*	*CD56*	*TTF1*
LX 101	59%	165200	No	N/A	N/A	SCLC	N/A	N/A	N/A
LX 102	65%	24700	Yes	95	0.77	SCLC	+	+	+
LX 103	66%	58080	Yes	132	3.79	SCLC	+	+	+
LX 104	75%	69000	Yes	102	0.68	SCLC	−	+	+
LX 105	75%	435000	Yes	91	0.31	SCLC	+	+	+
LX 106	85%	1487500	Yes	90	11.12	SCLC	+	+	+
LX 107	75%	18750	Yes	130	0.44	SCLC	+	+	+
LX 108	85%	318750	Yes	63	1.60	SCLC	+	+	+
LX 109	85%	212500	Yes	69	2.57	LCNEC	−	+/−	+/−
LX 110	50%	180000	Yes	83	3.18	SCLC	+	+	+
LX 111	66%	115500	Yes	188	2.03	SCLC	+	+	+
LX 112	66%	115500	No	N/A	N/A	SCLC	N/A	N/A	N/A

PDX  =  patient derived xenograft at passage 2 in nude mice; Purity  = **%** of nucleated cells that were tumor cells; SCLC  =  small cell lung cancer; LCNEC  =  large cell neuroendocrine carcinoma; N/A  =  not available; H&E  =  section stained with haematoxylin and eosin; Results of PDX section stained with antibodies against Synaptophysin (Synapto), CD56 and Thyroid Transcription Factor 1 (TTF1) are also shown.

### Targeted re-sequencing

In order to determine the effects of engraftment and passage on the genomic integrity of our PDX models, we employed a targeted, next-generation sequencing strategy to identify exonic mutations in the primary EBUS sample and its derivative passage-2 xenograft. Genomic DNA from both samples was analysed using the Ion Ampliseq Comprehensive Cancer Panel sequenced on the Ion Torrent platform (Life Technologies). This system amplifies and sequences the coding exons of 409 genes in which driver mutations have been identified.

The number of mapped sequencing reads ranged from 16 to 26 million, most of them on target (over 97% in all samples), with a minimum sequence depth of coverage in the targeted regions of 1000 times (Table S1). High uniformity of coverage was observed for all samples, ranging from 85 to 92% (Table S1). The total number of variants detected for each primary sample is shown in Table 3, along with the total number of coding region variants. The number of coding region variants in each sample shared with each corresponding xenograft ranged 55-92% (mean 81.4%). The entire set of variant calls for each sample is included as an excel spreadsheet in File S1.

**Table 3:** Variants detected in primary samples derivative xenografts.

	Total Variants			Coding Region Variants		
Sample	*Primary*	*Xenograft*	*%*	*Primary*	*Xenograft*	*%*
LX102	1266	1076	85	720	626	87
LX103	1128	666	59	628	377	60
LX104	1235	679	55	692	381	55
LX105	1151	1047	91	692	630	91
LX106	1172	1055	90	649	597	92
LX107	1167	899	77	671	544	81
LX108	1093	951	87	620	552	89
LX109	1163	1047	90	655	603	92
LX110	1329	1050	79	723	600	83
LX111	1345	1076	80	737	619	84

**Figure 4:**
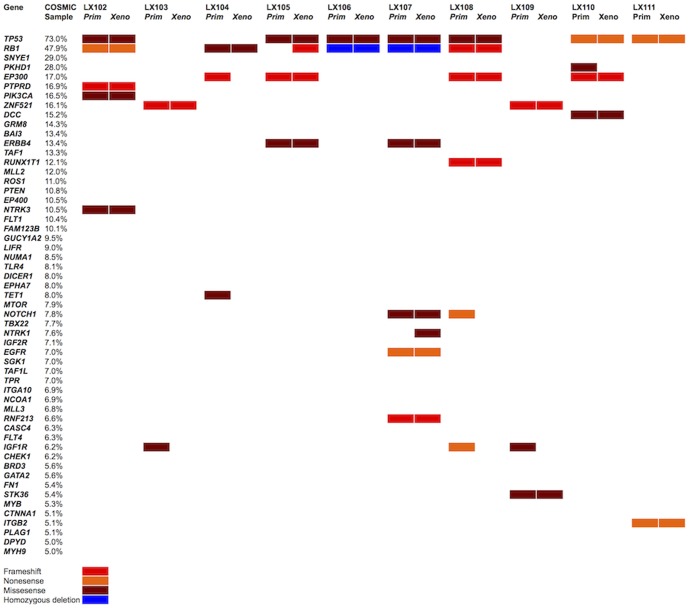
Ideogram summarising variants predicted to result in mutations commonly seen in SCLC. Genes are ranked by % according to their prevalence in the COSMIC database. Mutations detected in both the primary sample and the xenograft are shown.

Significant variants were identified as those predicted to (i) result in frameshift, nonsense or essential splice site mutations; or (ii) missense variants predicted to impair protein function with a SIFT [17] score ≤0.05. Shared coding region variants were then filtered using the following exclusion criteria: (i) known germline SNPs (ii) likely incidental mutations (http://mutagenetix.utsouthwestern.edu); and (iii) variants in genes commonly mutated in cancer that are likely to be of no functional significance [18]. These variants are listed in Table S2. We next ranked the affected genes according to their mutation frequency in the COSMIC database (http://cancer.sanger.ac.uk/cancergenome/projects/cosmic) with a reported incidence ≥5%. As shown in Figure 4, when common driver mutations were present in the primary sample, they were conserved in the corresponding xenograft. Mutations present only in the primary sample (*IGFR1*, *TET1*, *MTOR*) and only in the xenograft (*RB1*, *NTRK1*) were observed in seven primary sample-xenograft pairs (Figure 4). Mutations in *KRAS*, more typical of lung adenocarcinoma, were not detected.

## Discussion

PDX models have recently emerged as a way of more accurately modelling therapeutic responses [5]–[7] outcome [12], [19] and as source of high quality material for next-generation sequencing [20]. Typically, the generation of lung cancer PDX lines has been limited to the use of surgically resected material as the source of viable tumor cells [19], [21], [22]. Since less than 20% of lung cancer patients undergo surgery, this approach limits the establishment of PDX lines to early stage lung cancers. Moreover, SCLC is almost never surgically resected, as emphasized by recent whole genome studies [23], [24]. In our initial description of three SCLC PDX lines, we sourced material from bronchoscopic biopsies in rare cases where endobronchial lesions could be easily identified [4], [20]. Recently, Hodgkinson *et al* [25] described the successful generation of 4 SCLC PDX lines from circulating tumor cells from 6 patients, emphasizing the aggressive nature of these tumors, as well the potential for generating tractable models from minimally invasive techniques.

To our knowledge, this is the first description of EBUS-TBNA samples as a platform for the generation of PDX lines. Since SCLC much more commonly presents as intra-thoracic lymphadenopathy or mediastinal mass, EBUS-TBNA can potentially provide engraftable samples from almost all patients, thus dramatically expanding the potential for preclinical modelling in this disease. The shortcomings of PDX models, especially the lack of a host immune system, are well described [5]–[7]. Nevertheless, increasing use of these models is driven by evidence that PDX lines retain key features of the primary tumor that are irreversibly lost in conventional tissue culture [4]–[7]. For example, SCLC PDX lines do not respond to single agent therapy with the BCL2 antagonist ABT737 in contrast to xenograft models derived from conventional SCLC cell lines [26]. Our data now show that SCLC PDX lines derived from EBUS-TBNA retain the characteristic features of the primary tumor.

An ongoing concern with respect to PDX models is their ability to maintain the genotype of the primary tumor. Given the relatively small numbers of cells obtained from EBUS-TBNA samples, we are unable to determine whether the PDX genotype represents expansion of a more aggressive subclone based on the genomic heterogeneity model [27], [28]. Although our data clearly show that well described driver mutations are preserved in our EBUS-TBNA PDX lines for at least 2 passages, discordance in the detection of several mutations suggests that the process of xenografting may select for genetically distinct subclones derived from highly heterogeneous primary samples. In once case (LX105), a mutation in *RB1* was seen only in the PDX line, indicating that some xenograft lines may be more useful as stand alone models, rather than as identical copies of the patient's original tumour.

The amount and quality of DNA available from the EBUS sample allows for detailed, high depth sequencing analysis in contrast the more limited comparisons that can be made between circulating tumor cells and derivate PDX lines [25]. Interestingly, the sample that generated the PDX line LX109, which grew as a LCNEC tumor, lacked mutations commonly seen in SCLC, suggesting that molecular analysis of EBUS-TBNA specimens may add to the precision of conventional pathologic and cytologic criteria. Since structural variants in genes such as *MYC*, *MYCN* and *SOX2* are well described in SCLC [23], [24], our approach to generating PDX lines from EBUS-TBNA specimens could also serve as a platform for more intensive interrogation using WGS analysis to determine the effects of xenografting on chromosomal instability in SCLC.

Our results also highlight the potential for EBUS-TBNA samples as a source of high-quality DNA for molecular pathology analysis. Several groups have shown the retrospective analysis of fixed EBUS-TBNA samples can be used to identify clinically actionable mutations in lung cancer [29]–[33], and that high quality DNA, RNA and protein can be obtained from these samples [34], [35]. Our data extend these studies by demonstrating that freshly isolated EBUS-TBNA samples can generate high-quality DNA suitable for massively parallel targeted re-sequencing that avoids formalin artefact, and that can be carefully controlled for stromal artefact.

We have shown that in SCLC, EBUS-TBNA is a practical and minimally invasive technique that can generate high-quality DNA for next-generation sequencing, and can be used as a source of viable tumor cells that engraft in immunodeficient mice with extremely high efficiency. Furthermore, these grafts retain important features of the primary tumor, including mutations that are characteristic of SCLC. These data suggest that EBUS-TBNA is a technique through which respiratory physicians and thoracic surgeons can generate samples that can form the basis for novel preclinical research, and ultimately, actionable molecular diagnosis in SCLC and other intra-thoracic malignancies.

## Supporting Information

Figure S1 **Features of specimen LX109 and its derivative xenograft.** **A**. Diff-Quick stained cytology smear of diagnostic EBUS-TBNA sample. Scale bar = 15 µm. **B**. Diff-Quick stained cytology smear of experimental EBUS-TBNA sample. Scale bar = 15 µm. **C**. Hematoxylin and eosin stained section of the diagnostic cell block. Scale bar = 30 µm. **D**. Diagnostic cell block stained for CD56. Scale bar = 30 µm. **E**. Hematoxylin and eosin stained section of the derivative xenograft. Scale bar = 300 µm. **F**. Hematoxylin and eosin stained section of the derivative xenograft. Scale bar = 30 µm. **G**. Section of the derivative xenograft stained for CD56. Scale bar = 30 µm. **H**. Section of the derivative xenograft stained for TTF1. Scale bar = 30 µm.
(TIF)

Table S1 **Summary of mapping statistics of NGS experiments.**
(DOC)

Table S2 **Functionally significant variants shared between primary sample and xenograft.**
(DOC)

File S1 **Unfiltered variant calls across all samples.**
(XLS)
