# Supplementary figures and images for: Genomic Characterisation of Small Cell Lung Cancer Patient-Derived Xenografts Generated from Endobronchial Ultrasound-Guided Transbronchial Needle Aspiration Specimens
Source: PLoS One. 2014 Sep 5;9(9):e106862. doi: 10.1371/journal.pone.0106862 (PMC4156408; doi:10.1371/journal.pone.0106862)

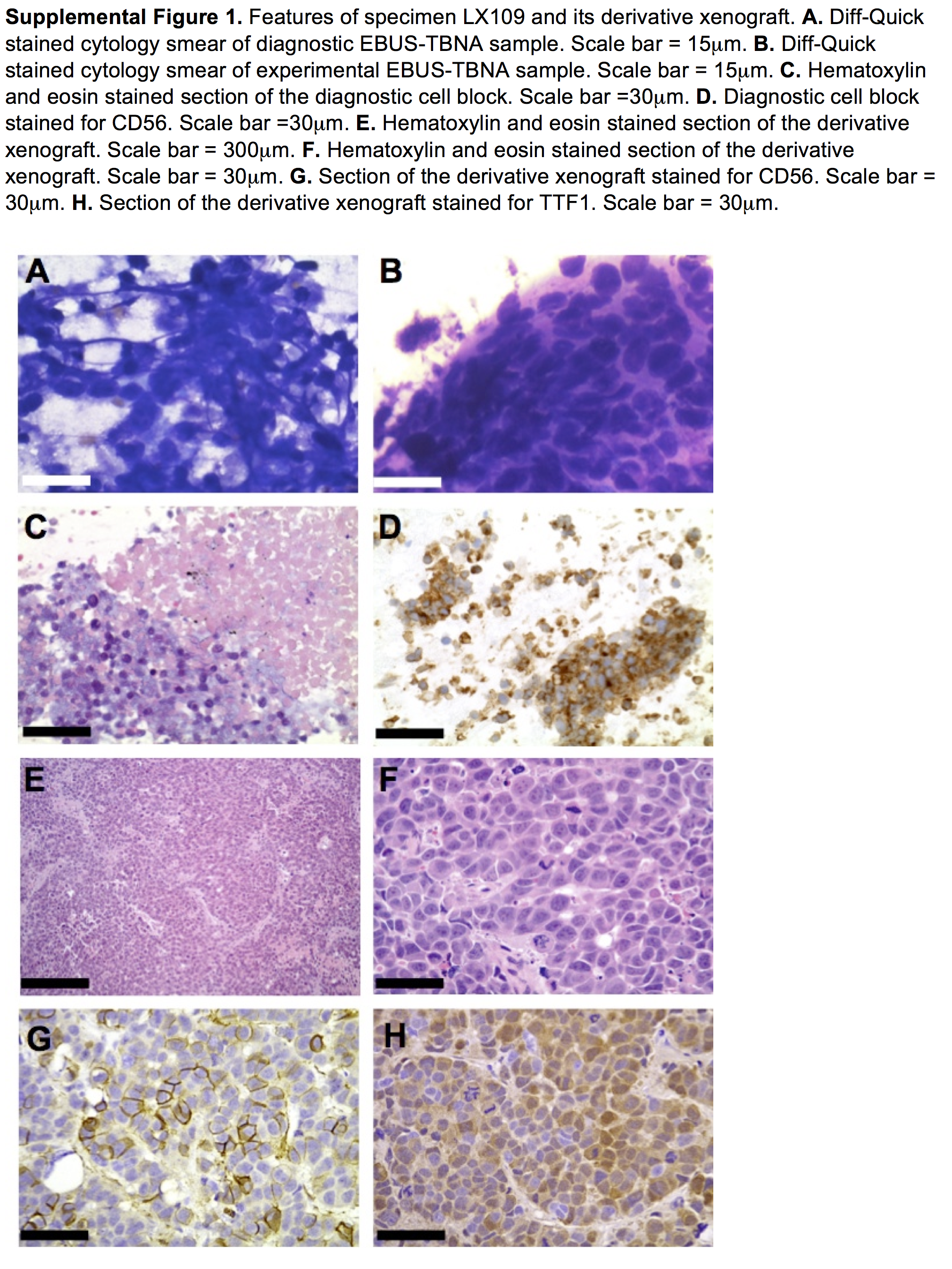

Supplement: Figure S1 — Features of specimen LX109 and its derivative xenograft. A. Diff-Quick stained cytology smear of diagnostic EBUS-TBNA sample. Scale bar = 15 µm. B. Diff-Quick stained cytology smear of experimental EBUS-TBNA sample. Scale bar = 15 µm. C. Hematoxylin and eosin stained section of the diagnostic cell block. Scale bar = 30 µm. D. Diagnostic cell block stained for CD56. Scale bar = 30 µm. E. Hematoxylin and eosin stained section of the derivative xenograft. Scale bar = 300 µm. F. Hematoxylin and eosin stained section of the derivative xenograft. Scale bar = 30 µm. G. Section of the derivative xenograft stained for CD56. Scale bar = 30 µm. H. Section of the derivative xenograft stained for TTF1. Scale bar = 30 µm. (TIF) [file pone.0106862.s001.tif]
