# Supplementary material for: Genomic Characterisation of Small Cell Lung Cancer Patient-Derived Xenografts Generated from Endobronchial Ultrasound-Guided Transbronchial Needle Aspiration Specimens
Source: PLoS One. 2014 Sep 5;9(9):e106862. doi: 10.1371/journal.pone.0106862 (PMC4156408; doi:10.1371/journal.pone.0106862)
Supplement: Table S1 — Summary of mapping statistics of NGS experiments. (DOC) [file pone.0106862.s002.doc]

**Table S1.** Summary of mapping statistics of NGS experiments.

|  | ***Primary*** |  |  |  |  | ***Xenograft*** |  |  |  |
| --- | --- | --- | --- | --- | --- | --- | --- | --- | --- |
| **Sample** | Mapped reads (x10^6^) | Reads on target (%) | Depth of coverage (mean) | Uniformity of coverage (%) |  | Mapped reads (x10^6^) | Reads on target (%) | Depth of coverage (mean) | Uniformity of coverage (%) |
| LX102 | 17.7 | 98.8 | 1,119 | 90.7 |  | 25.4 | 97.2 | 1,598 | 91.6 |
| LX103 | 23.1 | 99.0 | 1,479 | 91.4 |  | 22.2 | 98.0 | 1,406 | 91.5 |
| LX104 | 28.2 | 98.1 | 1,799 | 91.3 |  | 25.0 | 97.1 | 1,570 | 91.0 |
| LX105 | 19.9 | 99.0 | 1,258 | 85.4 |  | 19.5 | 98.5 | 1,245 | 92.0 |
| LX106 | 18.9 | 98.7 | 1,198 | 92.9 |  | 23.1 | 97.9 | 1,459 | 91.7 |
| LX107 | 23.6 | 99.1 | 1,512 | 90.1 |  | 22.6 | 98.5 | 1,433 | 92.3 |
| LX108 | 22.5 | 98.6 | 1,422 | 91.8 |  | 26.5 | 98.1 | 1,668 | 91.7 |
| LX109 | 20.0 | 98.3 | 1,259 | 92.0 |  | 19.8 | 98.0 | 1,249 | 92.7 |
| LX110 | 17.0 | 98.4 | 1,047 | 91.8 |  | 19.2 | 98.8 | 1,214 | 92.6 |
| LX111 | 18.4 | 98.7 | 1,156 | 91.2 |  | 24.0 | 98.6 | 1,526 | 92.0 |
