# Supplementary material for: Genomic Characterisation of Small Cell Lung Cancer Patient-Derived Xenografts Generated from Endobronchial Ultrasound-Guided Transbronchial Needle Aspiration Specimens
Source: PLoS One. 2014 Sep 5;9(9):e106862. doi: 10.1371/journal.pone.0106862 (PMC4156408; doi:10.1371/journal.pone.0106862)
Supplement: Table S2 — Functionally significant variants shared between primary sample and xenograft. (DOC) [file pone.0106862.s003.doc]

|  |  |  |  |  |  |  |  |  |  |
| --- | --- | --- | --- | --- | --- | --- | --- | --- | --- |
|  |  |  |  |  |  |  |  |  |  |
|  |  |  |  |  |  |  |  |  |  |
|  |  |  |  |  |  |  |  |  |  |
|  |  |  |  |  |  |  |  |  |  |
|  |  |  |  |  |  |  |  |  |  |
|  |  |  |  |  |  |  |  |  |  |
|  |  |  |  |  |  |  |  |  |  |
|  |  |  |  |  |  |  |  |  |  |
|  |  |  |  |  |  |  |  |  |  |
|  |  |  |  |  |  |  |  |  |  |
|  |  |  |  |  |  |  |  |  |  |

**Table S2.** Functionally significant variants shared between primary sample and xenograft.
